# Transcriptome analysis of *Pennisetum americanum* × *Pennisetum purpureum* and *Pennisetum americanum* leaves in response to high-phosphorus stress

**DOI:** 10.1186/s12870-024-05339-3

**Published:** 2024-07-06

**Authors:** Lili Zhao, Xin Zhao, Lei Huang, Xiaoyan Liu, Puchang Wang

**Affiliations:** 1 College of Animal Science, Guizhou University, Guiyang, 550025 China; 2 School of Life Sciences, Guizhou Normal University, Guiyang, 550001 China

**Keywords:** High-phosphorus stress, *Pennisetum americanum* × *Pennisetum purpureum*, *Pennisetum americanum*, Transcriptomics

## Abstract

Excessive phosphorus (P) levels can disrupt nutrient balance in plants, adversely affecting growth. The molecular responses of *Pennisetum* species to high phosphorus stress remain poorly understood. This study examined two *Pennisetum* species, *Pennisetum americanum* × *Pennisetum purpureum* and *Pennisetum americanum*, under varying P concentrations (200, 600 and 1000 µmol·L^− 1^ KH_2_PO_4_) to elucidate transcriptomic alterations under high-P conditions. Our findings revealed that *P. americanum* exhibited stronger adaption to high-P stress compared to *P. americanum*× *P. purpureum*. Both species showed an increase in plant height and leaf P content under elevated P levels, with *P. americanum* demonstrating greater height and higher P content than *P. americanum*× *P. purpureum*. Transcriptomic analysis identified significant up- and down-regulation of key genes (e.g. *SAUR*, *GH3*, *AHP*, *PIF4*, *PYL*, *GST*, *GPX*, *GSR*, *CAT*, *SOD1*, *CHS*, *ANR*, *P5CS* and *PsbO*) involved in plant hormone signal transduction, glutathione metabolism, peroxisomes, flavonoid biosynthesis, amino acid biosynthesis and photosynthesis pathways. Compared with *P. americanum*× *P. purpureum*, *P. americanum* has more key genes in the KEGG pathway, and some genes have higher expression levels. These results contribute valuable insights into the molecular mechanisms governing high-P stress in *Pennisetum* species and offer implications for broader plant stress research.

**Supplementary Information:**

The online version contains supplementary material available at 10.1186/s12870-024-05339-3.

## Introduction

Phosphorus (P), a vital nutrient in plant growth and development, constitutes a key element in nucleic acids, phospholipids, and high-energy phosphate compounds. It plays a crucial role in energy transfer, information transduction, photosynthesis, enzymatic reactions, and material metabolism [1, 2]. The availability of active P in soil and the efficiency of its uptake by plants significantly influence crop yield and quality. Although soil typically contains 500 to 2000 mg·kg^− 1^ of total P, the concentration of P that plants can effectively absorb is strikingly low, ranging from 0.1 to 10 µmol·L^− 1^ [3, 4]. Insufficient P uptake hampers cell division and proliferation, hindering the formation of new organs, which in turn adversely impacts plant physiological metabolism and growth, ultimately leading to a substantial reduction in crop yield and quality [5]. Conversely, over-application of P fertilizers can induce phosphorus toxicity in plants, diminishing the yield and quality of agricultural produce [6, 7]. The exact mechanisms underlying P toxicity remain elusive, though it is widely believed that elevated P levels contribute to deficiencies in iron, zinc, and copper [8, 9]. The impact of high P on plant nutrient uptake represents a significant aspect of plant stress [10]. Therefore, deciphering the physiological and molecular mechanisms that govern efficient P uptake and utilization in plants is critical for the genetic enhancement of plant P efficiency.

The morphological adaptations and physiological responses of plants to P stress have been extensively documented. Low P stress markedly diminishes photosynthesis in the aerial parts of plants, disrupts in vivo metabolism, retards growth and development, and leads to decreased plant height, leaf area, and the number of branches or tillers [11]. Previous studies have demonstrated that under low P stress in *Arabidopsis* [12], rice [13, 14], and soybean [15], there is an increase in osmoregulatory substances and protective enzymes such as proline, alongside a significant enhancement in the activities of ACP, SOD, POD, and CAT. These adaptations help scavenge superoxide radicals produced under stress and maintain the redox balance within and outside the cells, ensuring normal plant growth. In contrast, high P stress leads to an increase in inorganic P content and the photorespiration to photosynthesis ratio in plant leaves, a decrease in net photosynthetic rate, an increase in dark respiration rate, and intensification of the photosynthetic lunch break phenomenon, all of which inhibit plant growth and development [16]. The molecular regulation of plants under P stress has also been a subject of study. Zhao et al. (2023) identified key pathways, such as photosynthesis, plant hormone signal transduction, and MAPK signaling, involved in the response to P deficiency in *Lotus corniculatus* [17]. They pinpointed specific genes (*MmPHT1; 5*, *MmPHO1*, *MmPAP1*, etc.) and transcription factors (from the WRKY and MYB families) as regulators in this process. Chen et al. (2021) discovered that the citric and glyoxalate cycle pathways were critical in regulating P deficiency in Chinese fir, analyzing the expression profiles of related Unigenes under varying P conditions [18]. Furthermore, the transcriptomic responses of maize [19] and tomato [20] to low P stress have been thoroughly investigated, identifying genes responsive to drought stress. These studies collectively enhance our understanding of the complex responses of plants to varying P levels and contribute to the broader knowledge of plant stress physiology.

*Pennisetum americanum* × *Pennisetum purpureum*, a perennial herb, and *Pennisetum americanum*, an annual herb, both from the grass family, are predominantly found in tropical and subtropical regions, with some presence in temperate areas. The *Pennisetum* genus is characterized by a robust root system, erect and tall stems, long and spreading leaves, superior photosynthetic efficiency, excellent tillering ability, rapid regeneration post-mowing, fast growth in favorable climates, and high biomass and yield. These attributes render *Pennisetum* highly versatile, finding utility in fodder production, soil ecological remediation, biomass energy generation, manure disposal, landscaping, and paper manufacturing [21, 22]. In production, *Pennisetum* genus are often planted with high water and fertilizer to ensure their high yield. This often leads to the problem of excessive application of phosphorus fertilizer. Existing research on *Pennisetum* predominantly addresses abiotic stresses such as drought and oxidative stress [23, 24], while research on phosphorus stress, especially high phosphorus is relatively scarce. This study, therefore, investigates *P. americanum* × *P. purpureum* and *P. americanum* under hydroponic conditions with varying P levels. The objective is to decipher the adaptation mechanisms and molecular responses of these species to different P regimes, particularly focusing on high P tolerance. This research aims to identify the genes involved in P stress resilience, thereby providing theoretical insights for the cultivation and management of *Pennisetum* species.

## Materials and methods

### Plant materials and experimental design

*P. americanum* × *P. purpureum* and *P. americanum* seeds, procured from the Grassland Science Laboratory at the College of Animal Science, Guizhou University, China, were selected for this study. Healthy and well-formed seeds of *Pennisetum* were disinfected with 75% ethanol for 5 min, followed by thorough rinsing with distilled water. The seeds were then placed in Petri dishes lined with two layers of filter paper and incubated at a constant temperature of 25 °C in a humidity-controlled incubator. After seven days of germination, uniformly developed *Pennisetum* seedlings were selected and initially cultured in 1/2 strength nutrient solution. After 15 days, the seedlings were transferred to a full nutrient solution for an additional 10 days.

The phosphorus (KH_2_PO_4_) levels in the nutrient solution were adjusted to 200 µmol·L^− 1^ for moderate P, 600 µmol·L^− 1^ for high P, and 1000 µmol·L^− 1^ for very high P stress [25], with each treatment replicated three times. The full nutrient solution comprised Hoagland nutrient solution mixed with Amon’s trace elements. The standard P levels were set at KH_2_PO_4_ 0.2 mmol·L^− 1^, with other components including K_2_SO_4_ 0.75 mmol·L^− 1^, MgSO_4_·7H_2_O 0.65 mmol·L^− 1^, Ca(NO_3_)_2_·4H_2_O 2 mmol·L^− 1^, EDTA-Fe 0.1 mmol·L^− 1^, and various trace elements. For the P stress treatments, the KH_2_PO_4_ levels were set at 200 µmol·L^− 1^, 600 µmol·L^− 1^, and 1000 µmol·L^− 1^, while K_2_SO_4_ concentrations were adjusted to 850 µmol·L^− 1^, 750 µmol·L^− 1^, and 550 µmol·L^− 1^, respectively, to maintain a consistent K^+^ concentration of 1700 µmol·L^− 1^.

After 28 days, the leaves of the seedlings were harvested, ensuring sample uniformity. These leaf samples were immediately flash-frozen in liquid nitrogen and stored at -80 °C for subsequent physiological and RNA-seq analysis. There were three biological replicates per treatment. The phosphorus treatments of *P. americanum* × *P. purpureum* were denoted as DL_200, DL_600, DL_1000, and those of *P. americanum* as YL_200, YL_600, and YL_1000, respectively. This experimental design aimed to elucidate the physiological and molecular responses of *Pennisetum* species to varying P levels, providing insights into their adaptation mechanisms under P stress.

### Measurements of plant height and P content

Plant height was quantified using a ruler. For phosphorus (P) content determination in *P. americanum* × *P. purpureum* and *P. americanum*, the dried aerial parts and roots were digested with H_2_SO_4_-H_2_O_2_. The molybdenum blue method [26] was employed for P quantification, with the final readings taken using a microplate reader. P uptake efficiency (Pu) and P utilization efficiency (PUE) were calculated using the formulas:

Pu (mg) = P content (mg·g^− 1^) × dry weight (mg).

PUE (mg·mg^− 1^) = dry weight per plant (mg) / Pu per plant (mg).

### Transcriptome sequencing and gene expression profiling

Total RNA was extracted from the leaves of *P. americanum* × *P. purpureum* and *P. americanum* post 28 days of P treatment using the Trizol reagent kit (Invitrogen, Carlsbad, CA, USA), according to the manufacturer’s instructions. RNA quality was assessed using an Agilent 2100 Bioanalyzer (Agilent Technologies, Palo Alto, CA, USA) and verified through RNase-free agarose gel electrophoresis. Eukaryotic mRNA was enriched using Oligo(dT) beads, fragmented using fragmentation buffer, and reverse-transcribed into cDNA using the NEBNext Ultra RNA Library Prep Kit for Illumina (NEB #7530, New England Biolabs, Ipswich, MA, USA). The double-stranded cDNA fragments underwent end repair, A-tailing, adapter ligation, and purification with AMPure XP Beads (1.0X). Size selection was conducted via agarose gel electrophoresis, followed by PCR amplification. The resulting cDNA library was sequenced on the Illumina Novaseq6000 platform by Gene Denovo Biotechnology Co. (Guangzhou, China).

### RNA-seq data analysis

To obtain high-quality clean reads, raw reads were filtered using fastp (version 0.18.0) to remove adapters and low-quality bases. Bowtie2 (version 2.2.8) mapped the reads to the ribosomal RNA (rRNA) database, with rRNA-mapped reads being removed. The remaining clean reads were used for assembly and gene abundance calculation, employing StringTie v1.3.1 in a reference-based approach. Expression abundance and variation were quantified using FPKM values calculated by RSEM software. DESeq2 software conducted differential expression analysis, with genes having a false discovery rate (FDR) below 0.05 and an absolute fold change ≥ 2 considered differentially expressed genes (DEGs).

Short Time-series Expression Miner (STEM) [27] is software for sequence clustering, comparison and visual expression in a short time. All differentially expressed genes of the two individuals (*P. americanum* × *P. purpureum* and *P. americanum*) as input file, all other parameters set to default values, and regulation trend significantly changed with P value < 0.05.

GO enrichment analysis mapped all DEGs to GO terms in the Gene Ontology database (http://www.geneontology.org/), with significant enrichment defined by a hypergeometric test. Pathway analysis of DEGs was conducted using the KEGG database (http://www.genome.jp/kegg/pathway.html), employing a similar statistical approach to identify significantly enriched pathways. This comprehensive analysis aimed to elucidate the molecular mechanisms underpinning the response of *Pennisetum* species to varying P levels.

### Quantitative real-time PCR validation of DEGs based on transcriptomic data

To validate the results obtained from RNA-seq, twenty DEGs were selected for quantitative real-time PCR (qRT-PCR) analysis. cDNA was synthesized from the same RNA samples that were used for transcriptome sequencing. The qRT-PCR was conducted using a CFX Connect™ Real-Time System (Applied Biosystems) along with an UltraSYBR mixture (CWBiotech). The thermocycler parameters included an initial denaturation at 95 °C for 10 min, followed by 40 cycles of denaturation at 95 °C for 15 s and annealing at 60 °C for 1 min, within a total reaction volume of 20 µL. For normalization purposes, the 18 S rRNA gene, a constitutively expressed gene in *Pennisetum*, was used as the reference gene. Each sample was subjected to three technical replicates to ensure the reliability of the results.

### Statistical analysis

Data collation and analysis were performed using WPS Office software and SPSS 25.0 software. A one-way ANOVA was employed for statistical analysis, and the least significant difference (LSD) method was utilized for multiple comparisons. Significance was established at *p* < 0.05. All presented data are means ± standard errors (SEs) from three replicates. Graphical representations of the data were plotted using Origin 2021 software. This comprehensive approach ensured the accuracy and reliability of the findings, contributing significantly to the understanding of gene expression dynamics in *Pennisetum* species under varying P conditions.

## Results

### Determination of P content and plant height

**Fig. 1:**
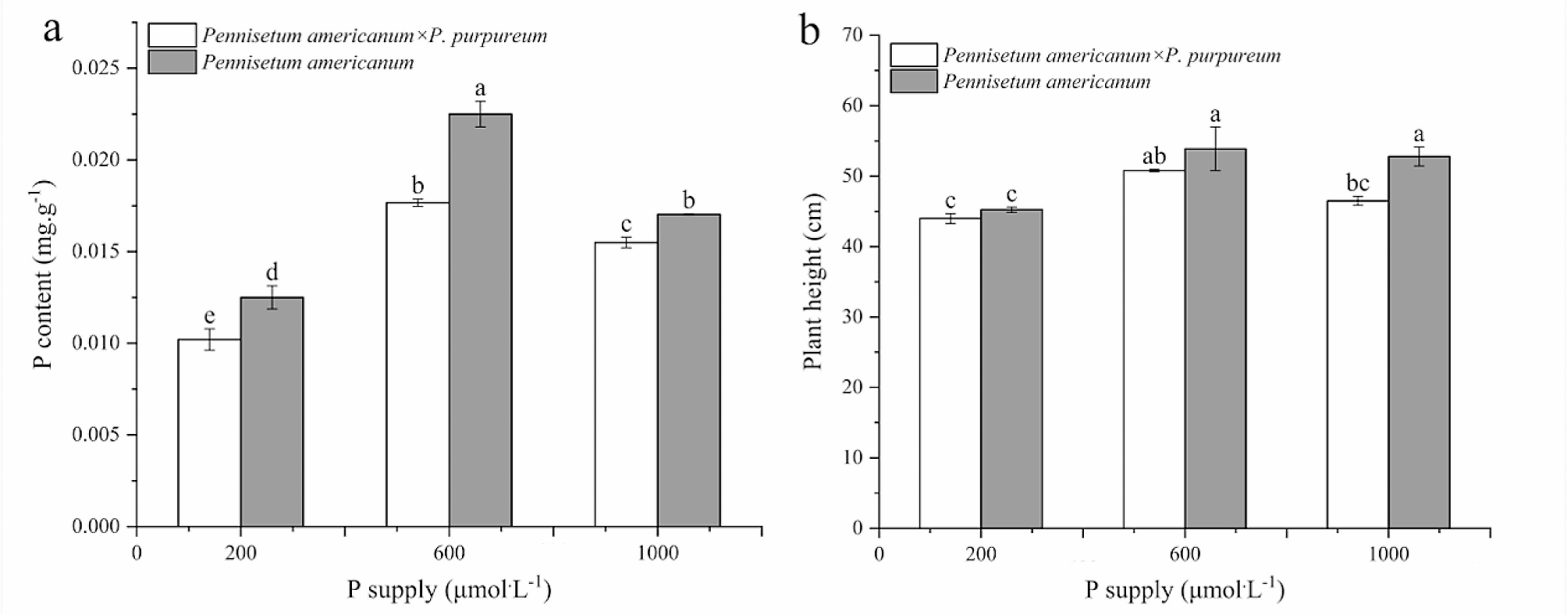
Effect of P supply on P content (**a**) and plant height (**b**) of *Pennisetum americanum×P. purpureum* and *P. americanum*. Data are means ± SE, *n* = 3; different lowercase letters above bars indicate significant differences (*P* < 0.05) between treatments

The study observed that both P content and plant height of *P. americanum* × *P. purpureum* and *P. americanum* varied with different levels of P supply (Fig. 1). An initial increase in these parameters was noted, followed by a decrease, with the peak for both P content and plant height observed at a P supply concentration of 600 µmol·L^− 1^ phosphorus (Pi). This phosphorus concentration is suitable for the growth of the experimental plant. Notably, compared to *P. americanum* × *P. purpureum*, *P. americanum* demonstrated a more pronounced increase in both P content and plant height across the varying levels of P supply.

### Identification of differentially expressed genes

**Fig. 2:**
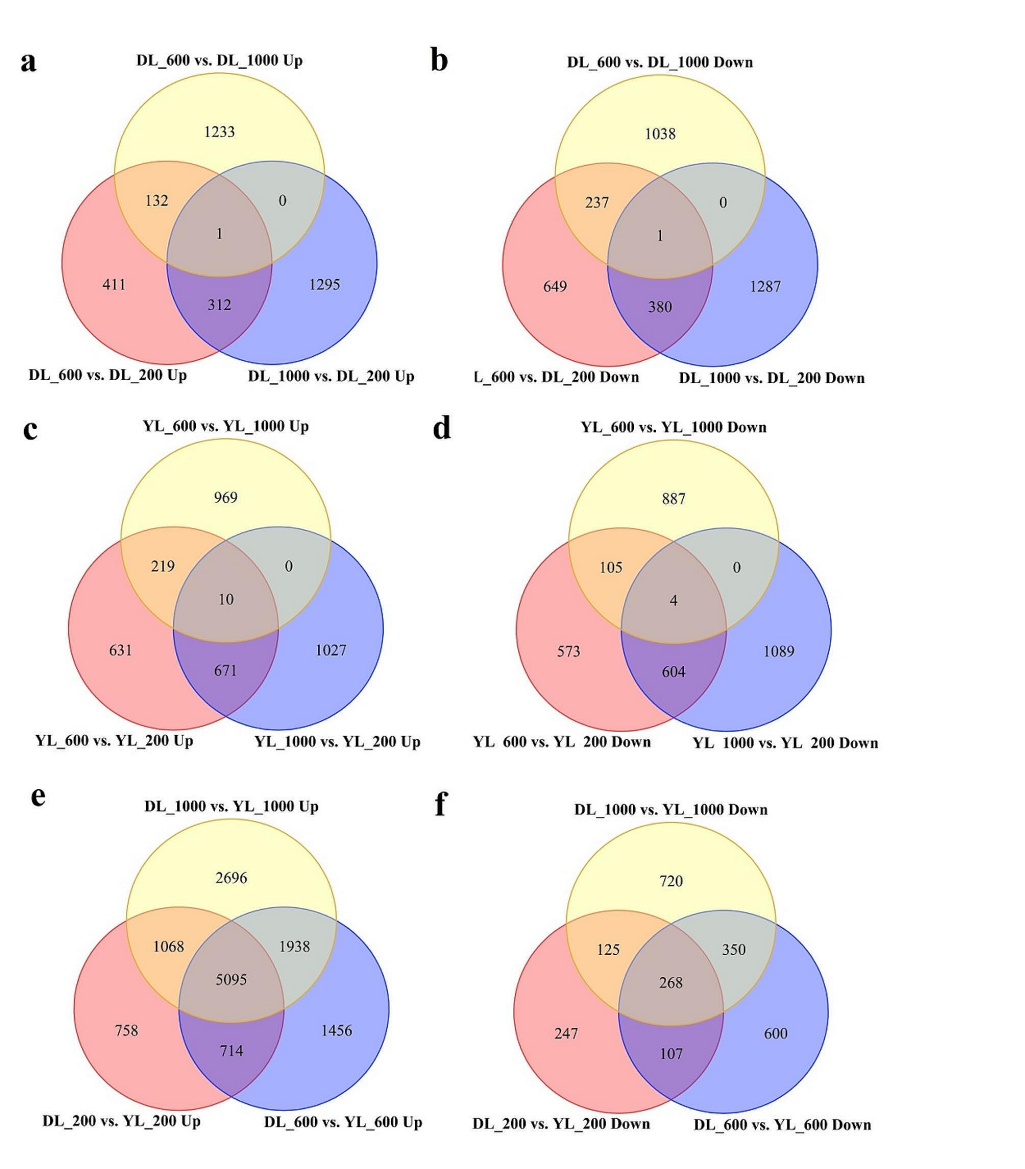
Venn diagrams of differentially expressed genes. Overlaps among up- (**a**) and downregulated genes (**b**) of *Pennisetum americanum×P. purpureum* under P supply conditions, up- (**c**) and downregulated genes (**d**) of *P. americanum* under P supply conditions, up- (**e**) and downregulated genes (**f**) of *Pennisetum americanum×P. purpureum* vs. *P. americanum* under P supply conditions

Eighteen cDNA libraries from the leaves of *P. americanum* × *P. purpureum* and *P. americanum*, cultivated under various P supply conditions for 28 days, were sequenced using Illumina high-throughput sequencing technology. This process generated a total of 119.9 Gbp of raw data, with individual samples yielding between 5.70 and 7.80 Gbp (Table S1). The sequencing data have been made publicly available in the NCBI database under the SRA accession number PRJNA870253. A total of 6976 DEGs were identified in *P. americanum* × *P. purpureum*, comprising 3384 upregulated and 3592 downregulated genes. In *P. americanum*, there were 6789 DEGs, with 3527 being upregulated and 3262 downregulated (Fig. 2). Notably, under the 600 µmol·L^− 1^ Pi condition, the numbers of DEGs, whether upregulated or downregulated, were lower than those at the 1000 µmol·L^− 1^ Pi level. A comparative analysis between *P. americanum* × *P. purpureum* and *P. americanum* under P supply conditions revealed a total of 16,142 DEGs, of which 13,725 were upregulated and 2417 were downregulated. The number of upregulated DEGs was higher than that of the downregulated ones (Fig. 2). These results highlight significant transcriptomic changes in response to different P supply levels in *Pennisetum* species. The differential expression patterns provide insights into the molecular mechanisms through which these plants adapt to varying P availability, potentially informing future research and breeding strategies for improved P efficiency in these and related species.

### Expression trend analysis of differential expression genes

**Fig. 3:**
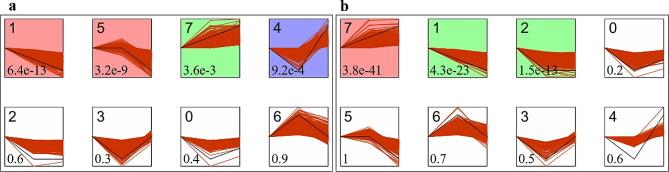
Expression trend profiles of differentially expressed genes of *P. americanum*×*P. purpureum* (**a**), *P. americanum* (**b**). Each box represents a different expression profile, colored profiles have a statistically significant number of genes assigned with *P* < 0.05, the upper left corner of the digital is profile ID, the lower left corner represents P value

The short Time-series Expression Miner (STEM) was used to further cluster the identified DEGs. Four colored profiles were significant in *P. americanum*×*P. purpureum* (Fig. 3a) and 3 profiles in *P. americanum* (Fig. 3b). In *P. americanum*×*P. purpureum*, profile 7 (872 genes) was up-regulated, profiles 1 (422 genes) and 5 (633 genes) were down-regulated and profile 4 (500 genes) was initially down-regulated and then up-regulated. In *P. americanum*, profile 7 (1147 genes) was upregulated, while profiles 1 (470 genes) and 2 (418 genes) were down-regulated.

### GO functional annotation enrichment analysis of differential expression genes

Gene Ontology (GO) analysis, a fundamental tool for gene functional annotation and enrichment analysis, was employed to categorize the functions of the predicted genes in *Pennisetum*. In the study, a significant number of DEGs were found to be associated with these GO categories under various P supply conditions. All significantly enriched DEGs were assigned to cellular components, biological processes and molecular functions. For DL_600 vs. DL_200, there were 27, 62 and 34 GO terms identified in these groups, among which structural constituent of ribosome, ribosome, structural molecule activity and ribosome biogenesis were the four most enriched subcategories (Table S2). For DL_1000 vs. DL_200, there were 28, 64 and 36 GO terms identified in these groups, respectively, among which oxidoreductase activity, structural constituent of ribosome, structural molecule activity and ribosome were the four most enriched subcategories (Table S2). For DL_600 vs. DL_1000, there were 30, 61 and 34 GO terms identified in these groups, among which DNA metabolic process, oxidoreductase activity, response to stress and homeostatic process were the four most enriched subcategories (Table S2). For YL_600 vs. YL_200, there were 30, 64 and 37 GO terms identified in these groups, among which oxidoreductase activity, transferase activity, transferring glycosyl groups, carbohydrate metabolic process and transmembrane transport were the four most enriched subcategories (Table S2). For YL_1000 vs. YL_200, there were 31, 65 and 36 GO terms were identified in these groups, respectively, among which oxidoreductase activity, carbohydrate metabolic process, ribosome and structural constituent of ribosome were the four most enriched subcategories (Table S2). For YL_600 vs. YL_1000, there were 29, 62 and 35 GO terms were identified in these groups, among which ribosome biogenesis, structural constituent of ribosome, ribosome and structural molecule activity were the four most enriched subcategories (Table S2). These findings indicate a diverse range of molecular pathways and processes affected by varying P supply levels in *Pennisetum* species. The distinct GO terms and subcategories enriched under different P conditions reflect the complex molecular responses of these plants to P availability, potentially contributing to the development of strategies for improving P use efficiency in *Pennisetum*.

### Differential expression genes pathway enrichment analysis

Kyoto Encyclopedia of Genes and Genomes (KEGG) pathway enrichment analysis is a valuable tool for understanding the biological functions of DEGs [28]. In our study, DEGs with a p-value ≤ 0.05 were considered significantly differentially expressed. Among the KEGG enrichment pathways of DL_600 vs. DL_200, DL_1000 vs. DL_200, DL_600 vs. DL_1000, YL_600 vs. YL_200, YL_1000 vs. YL_200, and YL_600 vs. YL_1000 under P supply conditions, 33, 18, 26, 39, 35 and 10 significantly enriched pathways (*p* < 0.05), respectively, were identified (Table S3). For DL_600 vs. DL_200, three significantly enriched pathways with a large number of DEGs included: ribosome (ko03010, 86 genes), phenylpropanoid biosynthesis (ko00940, 19 genes), and glutathione metabolism (ko00480, 14 genes) (Table S3). For DL_1000 vs. DL_200, amino sugar and nucleotide sugar metabolism (ko00520, 30 genes), toxoplasmosis (ko05145, 27 genes), and plant-pathogen interaction (ko04626, 23 genes) were the three main pathways (Table S3). For DL_600 vs. DL_1000, photosynthesis - antenna proteins (ko00196, 21 genes), starch and sucrose metabolism (ko00500, 13 genes), and pertussis (ko05133, 12 genes) were the three main pathways (Table S3). For YL_600 vs. YL_200, three significantly enriched pathways with a large number of DEGs included: carbon metabolism (ko01200, 42 genes), biosynthesis of amino acids (ko01230, 36 genes), and phenylpropanoid biosynthesis (ko00940, 33 genes) (Table S3). For YL_1000 vs. YL_200, the main pathway were ribosome (ko03010, 87 genes), carbon metabolism (ko01200, 48 genes), and biosynthesis of amino acids (ko01230, 48 genes) (Table S3). For YL_600 vs. YL_1000, ribosome (ko03010, 60 genes), plant-pathogen interaction (ko04626, 23 genes), and carbon fixation in photosynthetic organisms (ko00710, 10 genes) were the three main pathways (Table S3). These results indicate that phosphorus supply significantly influences a variety of metabolic and biosynthetic pathways in *P. americanum* × *P. purpureum* and *P. americanum*. The enrichment in pathways such as ribosome biogenesis, phenylpropanoid biosynthesis, and carbon metabolism underlines the complex response of these plants to varying P levels.

### DEGs related to plant hormone signal transduction

**Fig. 4:**
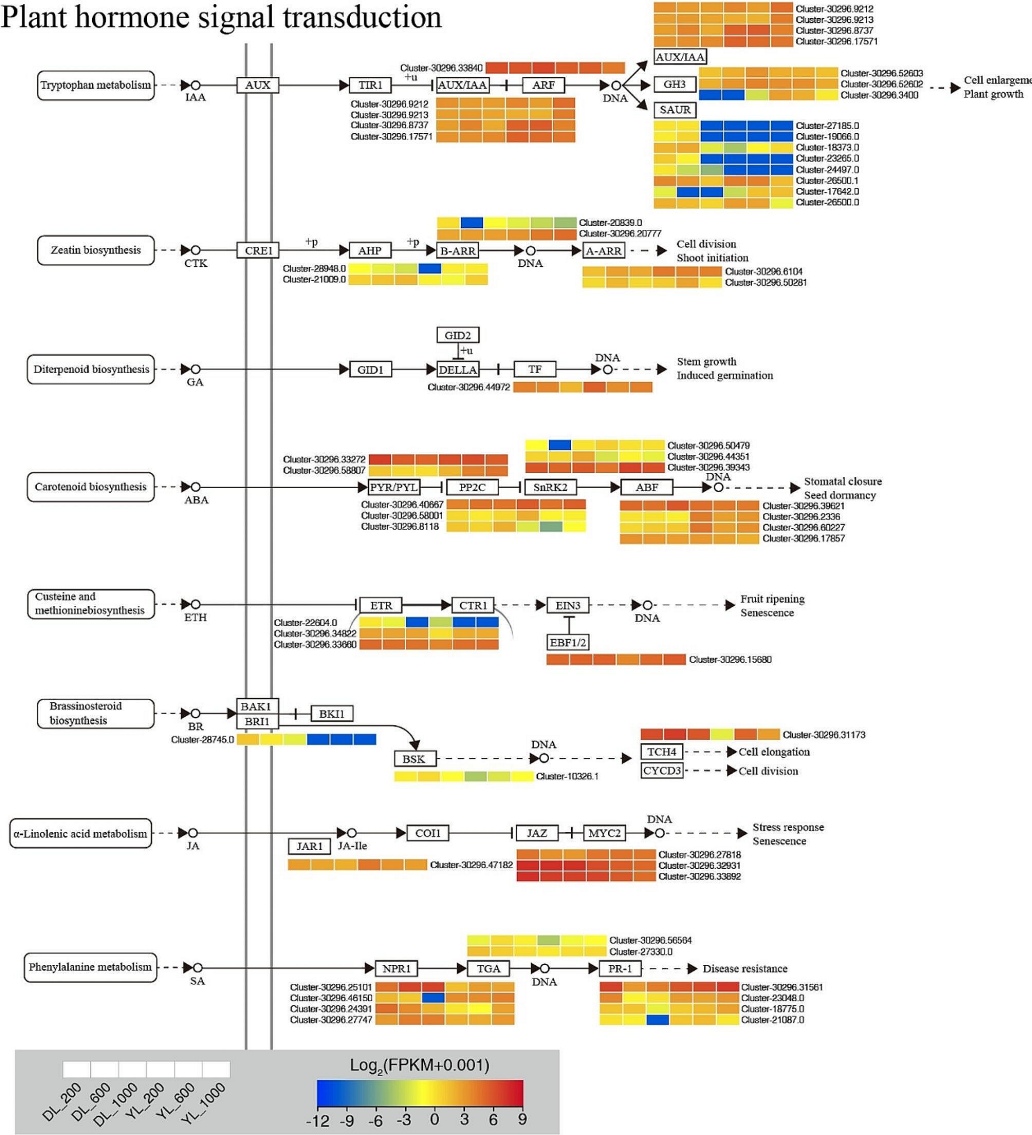
Plant hormone signal transduction-related genes identified in the RNA-seq that showed expression changes under P supply conditions. The color bar indicates the expression levels [represented as log_2_ (FPKM + 0.001)]; red indicates high expression level, blue indicates low expression level

The study identified a total of 56 DEGs associated with plant hormone signal transduction pathways, encompassing auxin, cytokinin, gibberellin, abscisic acid, ethylene, brassinosteroid, jasmonic acid, and salicylic acid (Fig. 4, Fig. S1 and Table S3). **In the auxin signaling pathway**, 5 *SAUR* genes were significantly downregulated, but 2 *GH3* genes were significantly upregulated in DL_1000 vs. DL_200. 3 *SAUR* genes were significantly upregulated in DL_600 vs. DL_1000. 1 *SAUR* gene was significantly upregulated in YL_600 vs. YL_200. 2 *SAUR*, 1 *GH3*, 1 *IAA* and 1 *ARF* genes were significantly downregulated, but 1 *SAUR* and 1 *AUX1* genes were significantly upregulated in YL_1000 vs. YL_200. 2 *SAUR*, 1 *GH3* and 1 *ARF* genes were significantly upregulated, but 2 *AUX1* genes were significantly downregulated in YL_600 vs. YL_1000. **In the cytokinin signaling pathway**, 1 *ARR-A* gene was significantly downregulated, but 1 *ARR-B* gene was significantly upregulated in DL_600 vs. DL_200. 1 *AHP* gene was significantly upregulated in YL_600 vs. YL_200. 1 *ARR-B* and 1 AHP genes were significantly upregulated in YL_1000 vs. YL_200. 1 *ARR-A* gene was significantly upregulated, but 1 *AHP* gene was significantly down in YL_600 vs. YL_1000. **In the gibberellin signaling pathway**, 1 *PIF4* gene was significantly downregulated in DL_1000 vs. DL_200, YL_600 vs. YL_200 and YL_1000 vs. YL_200, while upregulated in DL_600 vs. DL_1000. **In the abscisic acid signaling pathway**, 1 *PYL* gene was significantly downregulated, but 1 SNRK2 gene was significantly upregulated in DL_1000 vs. DL_200. 1 SNRK2 and 1 *ABF* genes were significantly downregulated in DL_600 vs. DL_1000. 1 *PYL* and 1 *SNRK2* genes were significantly upregulated, but 2 *ABF* and 2 *PP2C* genes were significantly downregulated in YL_600 vs. YL_200. 1 *PYL* and 1 *SNRK2* genes were significantly upregulated, but 2 *ABF* and 1 *PP2C* genes were significantly downregulated in YL_1000 vs. YL_200. 2 *PP2C* genes were significantly downregulated in YL_600 vs. YL_1000. **In the ethylene signaling pathway**, 1 *ETR* gene was significantly downregulated in DL_1000 vs. DL_200. 1 *BSK* gene was significantly upregulated in DL_600 vs. DL_1000. 1 *ETR* gene was significantly upregulated in YL_600 vs. YL_200. 1 *EBF1_2* genes were significantly upregulated in YL_1000 vs. YL_200. **In the brassinosteroid signaling pathway**, 1 *BRI1* gene was significantly downregulated in DL_1000 vs. DL_200. *1 TCH4* gene was significantly upregulated in YL_1000 vs. YL_200. **In the jasmonic acid signaling pathway**, 1 *JAZ* gene was significantly downregulated in DL_1000 vs. DL_200. 2 JAZ and 1 TAR1 genes were significantly downregulated in YL_1000 vs. YL_200. **In the salicylic acid signaling pathway**, 1 *TGA* and 1 *NPR1* genes were significantly upregulated, but 2 PR1 genes were significantly downregulated in DL_600 vs. DL_200. 1 *TGA*, 4 *PR1* and 2 *NPR1* genes were significantly downregulated, but 2 *NPR1* genes were significantly upregulated in DL_1000 vs. DL_200. 1 *PR1* and 1 *NPR1* genes were significantly upregulated in DL_600 vs. DL_1000. 1 *NPR1* gene was significantly downregulated in YL_600 vs. YL_1000. These results indicate a complex interplay of various plant hormone signaling pathways under different P supply conditions, highlighting the intricate regulatory mechanisms at play in response to P availability in *Pennisetum* species.

### DEGs related to physiological traits

**Fig. 5:**
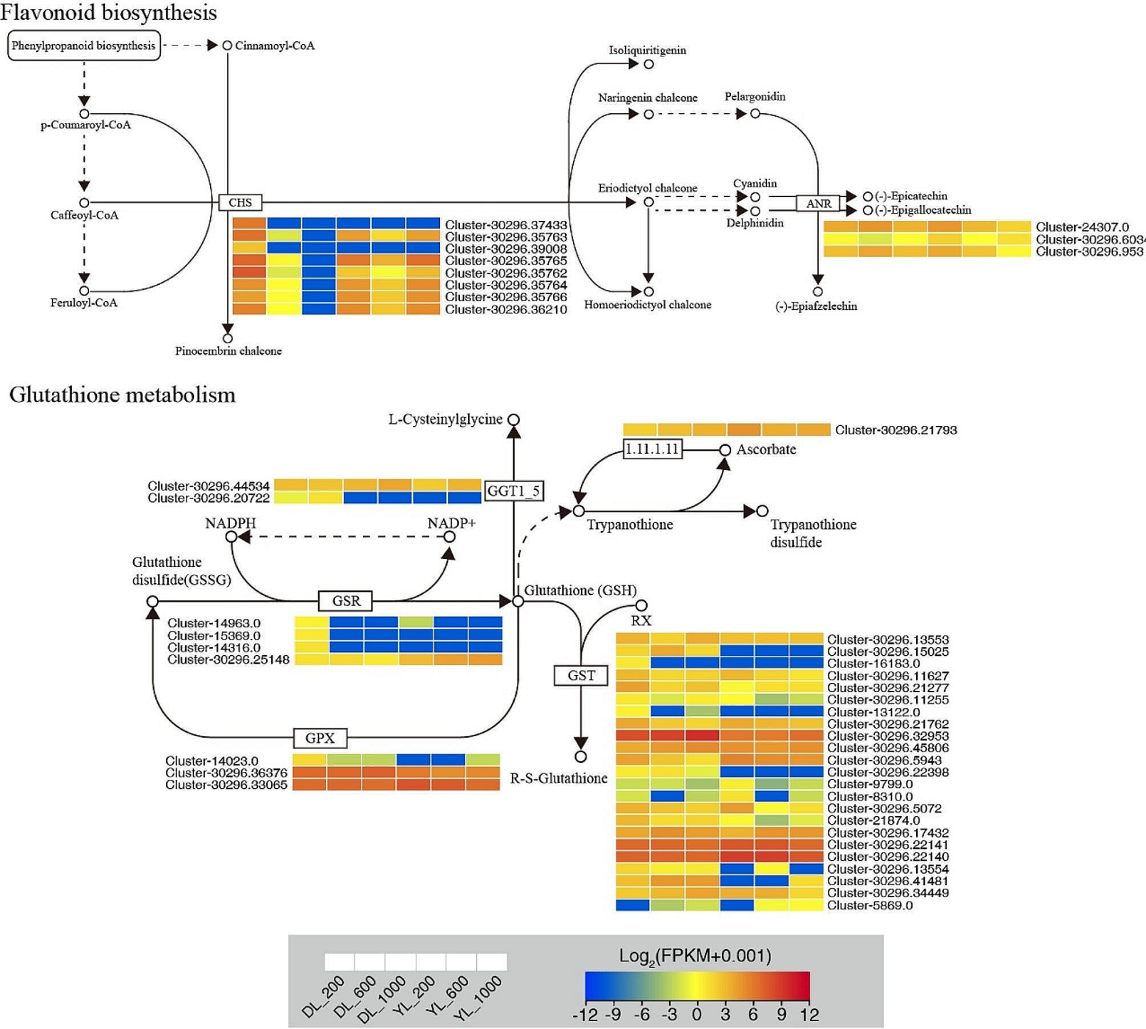
The pathways of flavonoid biosynthesis and glutathione metabolism identified in the RNA-seq that showed expression changes under P supply conditions. The color bar indicates the expression levels [represented as log_2_ (FPKM + 0.001)]; red indicates high expression level, blue indicates low expression level

**Fig. 6:**
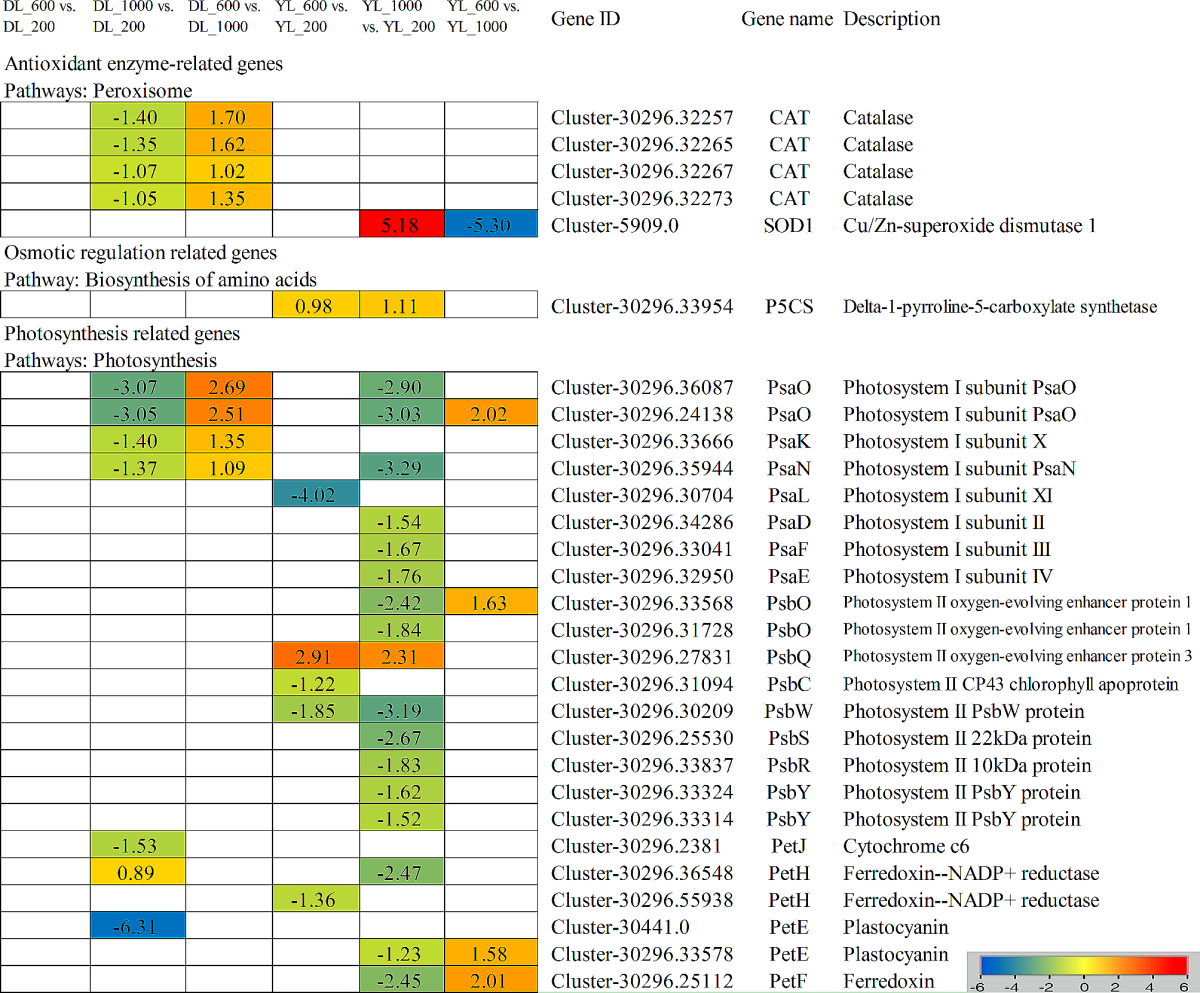
Antioxidant enzyme-related genes, Osmotic regulation related genes and Photosynthesis related genes identified in the RNA-seq that showed expression changes under P supply conditions. The log_2_[fold-change (FC)] colour scale ranges from − 6 to 6, with blue indicating downregulation and red indicating upregulation (see the colour set scale in the bottom left corner)

In *P. americanum* × *P. purpureum* and *P. americanum*, numerous DEGs related to physiological traits were identified, notably within pathways of amino acid biosynthesis, glutathione metabolism, peroxisomes, flavonoid biosynthesis, and photosynthesis (Fig. 5, Fig. S2 and Fig. 6). **44 oxidative stress-related genes**, 5 glutathione S-transferase (*GST*), 1 glutathione peroxidase (*GPX*), 3 glutathione reductase (*GSR*) and 5 chalcone synthase (*CHS*) genes were significantly downregulated, but 1 *GST* gene was significantly upregulated in DL_600 vs. DL_200. 4 *GST*, 1 *GPX*, 3 *GSR*, and 8 *CHS* genes were significantly downregulated, but 2 *GST* and 1 L-ascorbate peroxidase (*E1.11.1.11*) genes were significantly upregulated in DL_1000 vs. DL_200. 2 *GST* genes were significantly downregulated, but 1 *GST*, 1 Glutathione hydrolase (*GGT1_5*), 1 anthocyanidin reductase (*ANR*) genes were significantly upregulated in DL_600 vs. DL_1000. 4 *GST*, 1 *GPX*, 1 *GGT1_5*, 5 *CHS* and 1 *ANR* genes were significantly downregulated, but 1 *GST* gene was significantly upregulated in YL_600 vs. YL_200. 4 *GST*, 1 *GPX* and 2 *ANR* genes were significantly downregulated, but 1 *GST*, and 1 *GSR* genes was significantly upregulated in YL_1000 vs. YL_200. 1 *GST* and 4 *GST* genes were significantly upregulated in YL_600 vs. YL_1000.

**Five peroxisome-related genes**, 4 catalase (*CAT*) genes were significantly upregulated in DL_600 vs. DL_1000, and u/Zn-superoxide dismutase 1 (*SOD1*) genes was significantly upregulated in YL_1000 vs. YL_200, but 4 *CAT* genes were significantly downregulated in DL_1000 vs. DL_200, and 1 *SOD1* genes were significantly downregulated in YL_600 vs. YL_1000. **1 osmotic regulation-related genes**, the δ-1-pyrroline-5-carboxylate synthetase (*P5CS*) was upregulated in YL_600 vs. YL_200 and YL_1000 vs. YL_200. **Twenty-three photosynthesis-related genes**, 2 photosystem I subunit PsaO (*PsaO*), 1 photosystem I subunit X (*PsaK*), 1 photosystem I subunit PsaN (*PsaN*), 1 cytochrome c6 (*PetJ*) and 1 plastocyanin (*PetE*) genes were significantly downregulated, but 1 Ferredoxin–NADP + reductase (*PetH*) gene was significantly upregulated in DL_1000 vs. DL_200. 2 *PsaO*, 1 *PsaK* and 1 *PsaN* genes were significantly upregulated in DL_600 vs. DL_1000. 1 photosystem I subunit XI (*PsaL*), 1 photosystem II CP43 chlorophyll apoprotein (*PsbC*), 1 photosystem II PsbW protein (*PsbW*) and 1 ferredoxin-NADP + reductase (*PetH*) were significantly downregulated, but 1 Photosystem II oxygen-evolving enhancer protein 3 (*PsbQ*) gene was significantly upregulated in YL_600 vs. YL_200. 2 *PsaO*, 1 *PsaN*, and Photosystem I subunit II (*PsaD*), 1 Photosystem I subunit III (*PsaF*), 1 Photosystem I subunit IV (*PsaE*), 2 Photosystem II oxygen-evolving enhancer protein 1 (*PsbO*), 1 Photosystem II PsbW protein (*PsbW*), 1 Photosystem II 22 kDa protein (*PsbS*), Photosystem II 10 kDa protein (*PsbR*), 2 Photosystem II PsbY protein (*PsbY*), 1 *PetH*, 1 *PetE*, 1 Ferredoxin (*PetF*) genes were significantly downregulated, but 1 *PsbQ* gene was significantly upregulated in YL_1000 vs. YL_200. 1 *PsaO*, 1 PsbO, 1 PetE and 1 Pet F genes were significantly upregulated in YL_600 vs. YL_1000. These findings reveal a complex interplay of genes associated with various physiological traits in response to different P supply conditions. The identified DEGs in osmotic regulation, oxidative stress response, and photosynthesis pathways highlight the intricate molecular mechanisms plants employ to adapt to P availability.

### RT‑qPCR validation

**Fig. 7:**
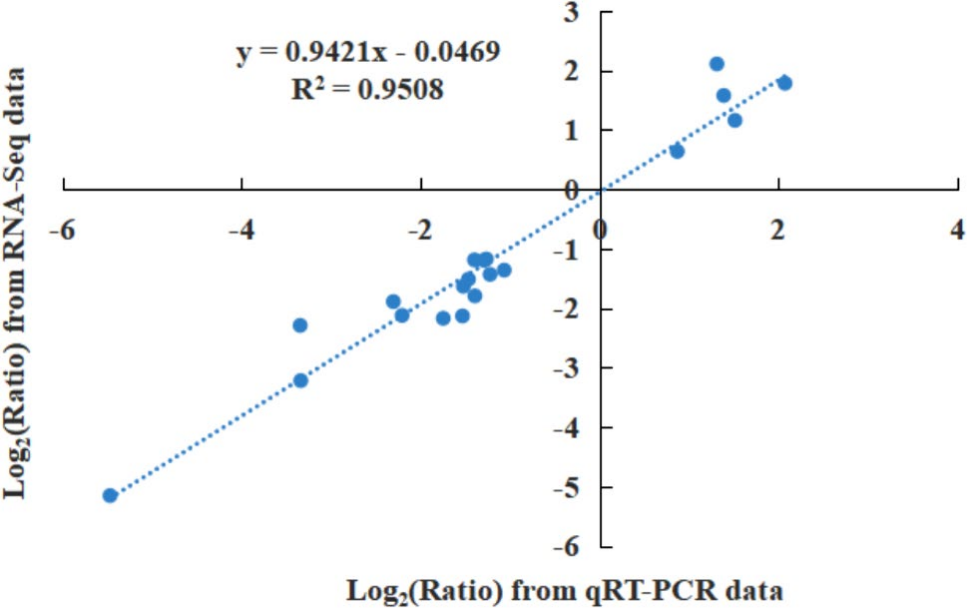
Scatter plot showing the changes in the expression [log_2_(FC)] of selected genes based on RNA-seq via qRT-PCR

For validation purposes, 20 differentially expressed genes (DEGs) were selected for qRT-PCR analysis, as detailed in Table S4 and illustrated in Fig. 7. The fragments per kilobase per million reads (FPKM) values from the RNA-seq data demonstrated a close correlation with their expression patterns observed in qRT-PCR, thereby confirming the reliability of the transcriptomic data.

## Discussion

To elucidate the molecular mechanisms governing plant responses to P stress, numerous prior investigations have employed transcriptome analysis, identifying several pivotal candidate genes and pathways implicated in the adaptation of plants to varying P concentrations. Notable candidate genes, including *SAUR*, *PP2C*, *JAZ*, *CAT1*, *SOD1*, *PSA*, *PSB*, *PFK1*, among others, have been pinpointed [17, 29, 30]. Furthermore, a series of crucial metabolic pathways encompassing plant hormone signal transduction, peroxisome function, photosynthesis, and carbon metabolism have been discerned. In the current study, we have also identified several key candidate genes—such as *SAUR*, *GH3*, *AHP*, *PIF4*, *PYL*, *GST*, *GPX*, *GSR*, *CAT*, *SOD1*, *CHS*, *ANR*, *P5CS*, and *PsbO*—pertaining to significant processes such as plant hormone signal transduction, glutathione metabolism, peroxisome function, flavonoid biosynthesis, amino acid biosynthesis, and photosynthesis.

Changes in leaf and root morphology and plant structural characteristics under P stress are intimately associated with alterations in endogenous hormone levels within plants [31–33]. In our investigation, genes associated with growth hormones, abscisic acid, cytokinins, ethylene, brassinosteroids, gibberellins, salicylic acid, and jasmonic acid signaling exhibited differential expression patterns in *P. americanum* × *P. purpureum*. Most of these genes demonstrated reduced expression levels when subjected to 1000 µmol.L^− 1^ Pi, with a notable exception being the genes linked to ethylene signaling, which displayed increased expression at 600 and 1000 µmol.L^− 1^ Pi in *P. americanum*. Our findings revealed an antagonistic relationship between auxin and cytokinin. This observation aligns with the study by Pacenza et al. (2021) [34], which established a connection between variations in *Arabidopsis thaliana* root morphology under low P stress and the activity of growth hormones in *Arabidopsis thaliana* hormone mutants. External application of auxin induced the formation of *Lupinus albus* cluster roots under normal P conditions, whereas the application of auxin translocation inhibitors hindered root development [35]. Additionally, reports of overexpression of cytokinin oxidase/dehydrogenase genes in tobacco and *Arabidopsis thaliana*, leading to cytokinin deficiencies and stunted plant height growth, suggest a negative impact of cytokinin on plant growth [36, 37]. In our study, the expression of SAUR genes associated with auxin signaling decreased in *P. americanum* × *P. purpureum* and *P. americanum* when exposed to 1000 µmol.L^− 1^ Pi, while GH3 gene expression increased in *P. americanum* × *P. purpureum* and decreased in *P. americanum*. IAA, AUX1, and ARF genes were exclusively expressed in *P. americanum*. *P. americanum* × *P. purpureum* exhibited the expression of ARR-A and ARR-B genes in cytokinin signaling at 1000 µmol.L^− 1^ Pi, and at 600 and 1000 µmol.L^− 1^ Pi, both ARR-A and ARR-B, along with AHP genes, were expressed. This suggests that higher P concentrations heighten plant sensitivity to auxin and cytokinin, influencing downstream gene expression levels and initiating responses to P stress. Gibberellin (GAs) is a pivotal hormone governing plant growth and development, particularly in regulating stem elongation [38, 39]. Changes in gibberellin synthesis, metabolism, or signal transduction can significantly impact plant height [40, 41]. In our study, the expression of the PIF4 gene in *P. americanum* × *P. purpureum* and *P. americanum* declined at 600 and 1000 µmol.L^− 1^ Pi compared to 200 µmol.L^− 1^ Pi, while plant height increased at 600 and 1000 µmol.L^− 1^ Pi. This observation suggests a potential negative regulation of gibberellin content by the PIF4 gene, although further analysis is warranted to establish the precise underlying mechanisms. Moreover, we observed differential up- or down-regulation of PYL, SNRK2, ABF, ETR, TCH4, JAZ, JAR1, TGA, PR1, and NPR1 genes associated with abscisic acid, ethylene, brassinosteroids, salicylic acid, and jasmonic acid signaling in both *P. americanum* × *P. purpureum* and *P. americanum* under high P stress. This underscores the involvement of these phytohormone-related genes in the plant’s response to phosphorus toxicity. Intriguingly, we noted a lower number of hormone-related genes in *P. americanum* × *P. purpureum* compared to *P. americanum*, and there are more upregulated genes in *P. americanum*, indicating that *P. americanum* exhibits greater adaptability to P stress, relying on more efficient hormonal regulation mechanisms.

It has been observed that plants can induce membrane lipid peroxidation through the generation of reactive oxygen species (ROS), consequently enhancing the permeability of plant cell membranes and resulting in cellular dysfunction under P stress [42]. The activity of antioxidant enzymes, including CAT, SOD, POD, and APX, plays a pivotal role in the plant’s antioxidant defense system during stressful conditions [13, 43]. In our study, we found that SOD, CAT, and APX activities increased and then decreased with increasing P concentration, and all of them were significantly higher in *P. americanum* than those in *P. americanum* × *P. purpureum*. At the same time, we noted a decrease in the expression of four CAT genes and increase in the expression of one E1.11.1.11 gene in *P. americanum* × *P. purpureum* as P concentration increased. Conversely, the SOD1 gene in *P. americanum* exhibited an increase in expression with rising P levels. These findings imply that SOD is responsible for the conversion of harmful substances into H_2_O_2_, which is subsequently transformed into O_2_ and H_2_O by antioxidant enzymes such as CAT. Furthermore, excess ROS are eliminated by APX, ultimately enhancing plant tolerance under P stress [44].

In our study, we identified oxidative stress-related pathways, particularly those related to glutathione metabolism, and observed alterations in the expression of genes associated with this metabolic pathway under P stress. Genes such as GPX, GST, GSR, and GGT1_5 are integral members of the antioxidant-related enzyme system [45]. Among these, GPX plays a crucial role in reducing H_2_O_2_ levels and lipid peroxidation through its involvement in glutathione metabolism, while GST contributes significantly to enhancing plant tolerance to abiotic stress by improving H_2_O_2_ scavenging and minimizing cytotoxicity under peroxide stress conditions [46]. In our investigation, the majority of GST, GPX, GSR, and GGT1_5 genes in both *P. americanum* × *P. purpureum* and *P. americanum* exhibited decreased expression as phosphorus concentration increased. This phenomenon may be attributed to the reduction of oxidized glutathione metabolites to reduced glutathione in response to increasing P levels, leading to the substantial accumulation of reduced glutathione. This accumulation, in turn, enhances *P. americanum*’s ability to scavenge H_2_O_2_ during abiotic stress and alleviates the negative effects of H_2_O_2_-induced stress [47].

Anthocyanins are known to exhibit scavenging properties against reactive oxygen species, and their biosynthesis involves the catalytic activity of several genes, including PAL, CHS, F3H, DFR, ANS, and others, within the phenylpropanoid and flavonoid biosynthetic pathways [48, 49]. In our current study, we observed a decrease in CHS gene expression in both *P. americanum* × *P. purpureum* and *P. americanum* under high P concentrations, while the ANR gene was exclusively expressed in *P. americanum*, albeit with reduced expression levels. While it is essential to note that gene expression alone may not fully capture the antioxidant properties of *P. americanum*, further validation in cellular and in vivo tissues is warranted. Nevertheless, these findings suggest a certain level of tolerance in *P. americanum*.

Plants can enhance their resistance to abiotic stress by accumulating substantial quantities of osmoregulatory substances, such as free proline, soluble proteins, and soluble sugars, to augment stress resilience, maintain cell membrane stability, and prevent excessive dehydration [50, 51]. In our study, we identified an osmoregulation-related gene, Delta-1-pyrroline-5-carboxylate synthetase (*P5CS*), associated with amino acid biosynthesis, which has been explored for its role in plant drought stress resistance. *P5CS* serves as the principal rate-limiting enzyme responsible for proline accumulation, and the increased expression of *P5CS* in plants contributes to their tolerance to abiotic stress [52, 53]. Notably, the P5CS gene was exclusively found in *P. americanum*, and its expression increased under high P concentrations. This suggests that *P. americanum* exhibits greater P stress tolerance relative to *P. americanum* × *P. purpureum*. This was consistent with the increase in proline content in *P. americanum*. *P. americanum*, in response to P stress, appears to adopt a protective strategy by elevating proline content to enhance water retention and uptake capacity, reflecting its need to maintain cellular turgor and combat the adverse effects of P stress.

Photosynthesis stands as the paramount source of plant biomass production, and its effectiveness is profoundly reliant on P-containing compounds. Phosphorus plays a pivotal role in facilitating photosynthesis [54]. In conditions of low P availability, it is customary for the expression of genes participating in photosynthetic processes, including those associated with photosystem I (PSI), photosystem II (PSII), and genes encoding subunits of vesicle-like membrane ATP synthases, to be typically suppressed [55, 56]. However, in our study, genes linked to the photosynthetic pathway in *P. americanum* × *P. purpureum* and *P. americanum* under elevated P concentrations were predominantly distributed among PSI protein genes (PsaO, PsaK, PsaN, PsaL, PsaD, PsaF, and PsaE), PSI protein genes (PsbO, PsbQ, PsbC, PsbW, PsbR, PsbS, and PsbY), as well as genes associated with photosynthetic electron transport (PetH, PetE, and PetF). Most of these genes exhibited down-regulated expression patterns, implying that higher phosphorus levels exert a repressive influence on the photosynthetic system.

Furthermore, there was more genes related to photosynthesis in *P. americanum* than in *P. americanum* × *P. purpureum*, indicating that *P. americanum* mobilized more genes to participate in regulatory adaptation under high phosphorus conditions. However, the downregulation of gene expression related to photosynthesis is more pronounced in *P. americanum* than in *P. americanum* × *P. purpureum*, and the reason for this remains to be further experimentally confirmed.

It is noteworthy that the phytohormone-related genes and various genes associated with physiological indicators may collectively participate in the regulation of morphological, metabolic, physiological, and biochemical changes in plants under conditions of high phosphorus stress through intricate and coordinated interactions.

## Conclusions

In conclusion, our study revealed that the elevated phosphorus uptake observed in leaf tissues at 28 days may be associated with a rapid activation of the transcriptome response. Moreover, we observed the plant height and leaf P content in *P. americanum* were significantly higher than those of *P. americanum* × *P. purpureum* when subjected to P concentrations of 600 and 1000 µmol.L^− 1^. This finding aligns with our screening results, indicating that compared to *P. americanum* × *P. purpureum*, *P. americanum* has a higher number of key genes in the KEGG pathway, and some genes have higher expression levels. Additionally, we identified genes related to hormones and physiological indicators that play crucial roles in the response to P stress. These genes are involved in significant pathways such as plant hormone signal transduction and glutathione metabolism. The transcriptome data generated from our study lay the foundation for future investigations into the mechanisms underlying plant responses to high P stress.

### Electronic supplementary material

Below is the link to the electronic supplementary material.

Supplementary Material 1

Supplementary Material 2

## Data Availability

All data supporting the findings were contained in the manuscript and its supplementary files and the RNA-seq raw data. And all the RNA-seq raw data were uploaded in the SRA of NCBI (https://www.ncbi.nlm.nih.gov/sra/, the accession number is PRJNA783425).
