# Supplementary material for: Transcriptome analysis of Pennisetum americanum × Pennisetum purpureum and Pennisetum americanum leaves in response to high-phosphorus stress
Source: BMC Plant Biol. 2024 Jul 6;24:635. doi: 10.1186/s12870-024-05339-3 (PMC11227232; doi:10.1186/s12870-024-05339-3)
Supplement: Supplementary file 2 — Supplementary Material 2 [file 12870_2024_5339_MOESM2_ESM.docx]

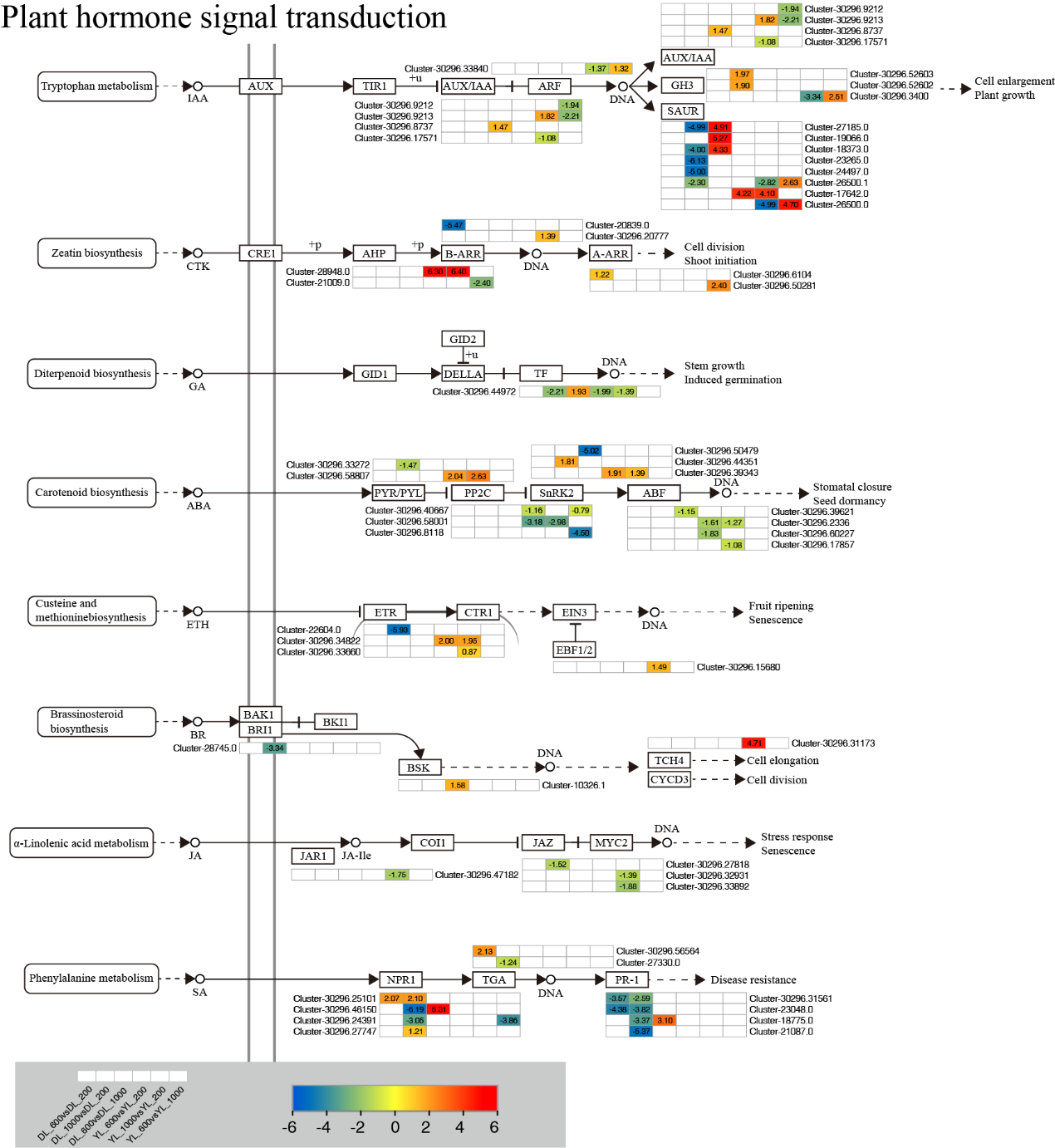


**Fig. S1** Plant hormone signal transduction-related genes identified in the RNA-seq that showed expression changes under P supply conditions. The log_2_[fold-change (FC)] colour scale ranges from −6 to 6, with blue indicating downregulation and red indicating upregulation (see the colour set scale in the bottom left corner).


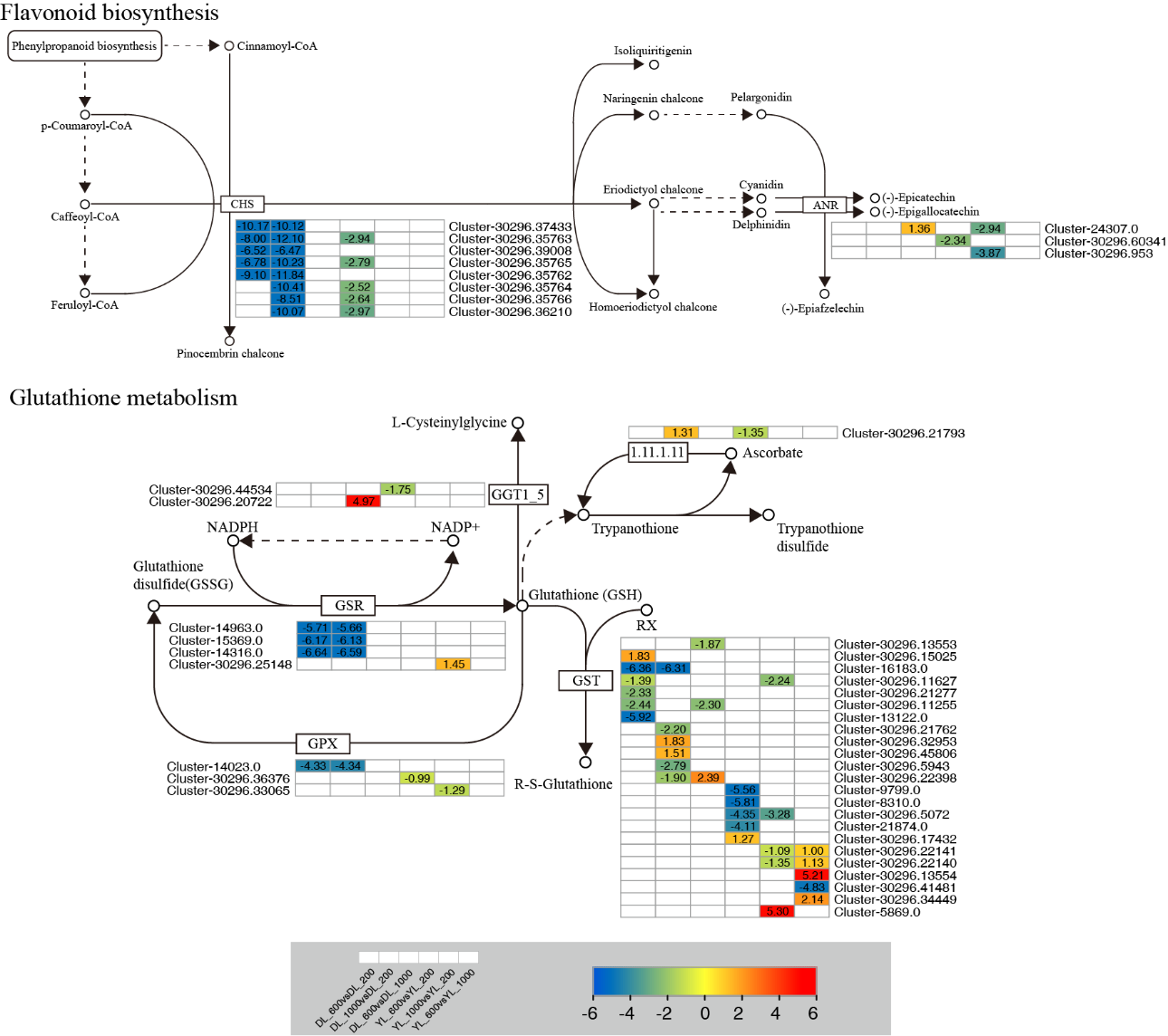


**Fig. S2** The pathways of flavonoid biosynthesis and glutathione metabolism identified in the RNA-seq that showed expression changes under P supply conditions. The log_2_[fold-change (FC)] colour scale ranges from −6 to 6 with blue indicating downregulation and red indicating upregulation (see the colour set scale in the bottom left corner).
